# Stereotactic Body Radiotherapy and Immunotherapy for Older Patients with Oligometastases: A Proposed Paradigm by the International Geriatric Radiotherapy Group

**DOI:** 10.3390/cancers15010244

**Published:** 2022-12-30

**Authors:** Nam P. Nguyen, Ahmed Ali, Vincent Vinh-Hung, Olena Gorobets, Alexander Chi, Thandeka Mazibuko, Natália Migliore, Maria Vasileiou, David Lehrman, Mohammad Mohammadianpanah, Seyed Alireza Javadinia, Gokoulakrichenane Loganadane, Trinanjan Basu, Satya Bose, Ulf Karlsson, Huan Giap

**Affiliations:** 1 Department of Radiation Oncology, Howard University, Washington, DC 20060, USA; 2 Division of Hematology Oncology, Howard University, Washington, DC 20060, USA; 3 Department of Radiation Oncology, Centre Hospitalier de la Polynesie Francaise, Papeete 98716, Tahiti, French Polynesia; 4 Department of Maxillofacial Surgery, Centre Hospitalier Universitaire de Martinique, 97213 Martinique, France; 5 Department of Radiation Oncology, Beijing Chest Hospital, Capital Medical University, Beijing 101149, China; 6 Department of Radiation Oncology, International Geriatric Radiotherapy Group, Washington, DC 20001, USA; 7 Barretos School of Health Sciences Dr. Paulo Prata, Barretos 14785-002, Sao Paulo, Brazil; 8 Department of Pharmacy, School of Health Sciences, National and Kapodistrian University of Athens, 15771 Athens, Greece; 9 Department of Radiation Oncology, Namazi Hospital, Shiraz University of Medical Sciences, Shiraz 71348-14336, Iran; 10 Non-Communicable Diseases Research Center, Sabzevar University of Medical Sciences, Sabzevar 96177-47431, Iran; 11 Department of Radiation Oncology CHU Mondor, University of Paris Est Creteil, 94000 Creteil, France; 12 Department of Radiation Oncology, HCG Cancer Center Borivali, and HCG ICS, Cobala, Mumbai 400092, India; 13 Department of Radiation Oncology, Medical University of South Carolina, Charleston, SC 29576, USA

**Keywords:** older, cancer patients, oligometastases, SBRT, CPI

## Abstract

**Simple Summary:**

Oligometastases carries a better prognosis. Even though standard treatment for distant metastases is systemic therapy, those with oligometastases have a better survival rate if they receive surgery or stereotactic body radiotherapy (SBRT). The advantage of SBRT over surgery is its minimal toxicity and the induction of program death ligand 1 (PD-L1) formation. Thus, SBRT may increase the tumor response to immunotherapy with checkpoint inhibitors (CPI). We propose a protocol using SBRT upfront for oligometastases, followed four to six weeks later by CPI for older cancer patients as they may not tolerate conventional chemotherapy. This hypothesis should be tested in future prospective clinical trials.

**Abstract:**

The standard of care for metastatic disease is systemic therapy. A unique subset of patients with limited metastatic disease defined as distant involvement of five anatomic sites or less (oligometastases) have a better chance of remission or improved survival and may benefit from local treatments such as surgery or stereotactic body radiotherapy (SBRT). However, to prevent further spread of disease, systemic treatment such as chemotherapy, targeted therapy, and hormonal therapy may be required. Older patients (70 years old or above) or physiologically frail younger patients with multiple co-morbidities may not be able to tolerate the conventional chemotherapy due to its toxicity. In addition, those with a good performance status may not receive optimal chemotherapy due to concern about toxicity. Recently, immunotherapy with checkpoint inhibitors (CPI) has become a promising approach only in the management of program death ligand 1 (PD-L1)-positive tumors. Thus, a treatment method that elicits induction of PD-L1 production by tumor cells may allow all patients with oligometastases to benefit from immunotherapy. In vitro studies have demonstrated that high dose of radiotherapy may induce formation of PD-L1 in various tumors as a defense mechanism against inflammatory T cells. Clinical studies also corroborated those observations. Thus, SBRT, with its high precision to minimize damage to normal organs, may be a potential treatment of choice for older patients with oligometastases due to its synergy with immunotherapy. We propose a protocol combining SBRT to achieve a minimum radiobiologic equivalent dose around 59.5 Gy to all tumor sites if feasible, followed four to six weeks later by CPI for those cancer patients with oligometastases. All patients will be screened with frailty screening questionnaires to identify individuals at high risk for toxicity. The patients will be managed with an interdisciplinary team which includes oncologists, geriatricians, nurses, nutritionists, patient navigators, and social workers to manage all aspects of geriatric patient care. The use of telemedicine by the team may facilitate patient monitoring during treatment and follow-up. Preliminary data on toxicity, local control, survival, and progression-free survival may be obtained and serve as a template for future prospective studies.

## 1. Introduction

Cancer patients with distant metastases at presentation frequently have a poor prognosis. Their survival depends on the type of cancer and response to systemic therapy. Older cancer patients (defined as 70 years old or above) in particular may have the worst outcomes because of frailty and the presence of co-morbidities which may not allow them to receive optimal chemotherapy [1]. However, among patients with a small number of metastatic sites (up to five) defined as oligometastases, long-term survival may be feasible with aggressive management of primary disease and all metastatic sites using systemic therapy and local therapies [2]. Surgery for older cancer patients with oligometastases carries a higher postoperative mortality and shorter survival compared to younger patients [3]. Thus, surgery may not be the preferred option for patients with multiple co-morbidities and is usually selected for patients with a good performance status [4]. On the other hand, stereotactic body radiotherapy (SBRT) is frequently well tolerated in older cancer patients with multiple co-morbidities which preclude them from having surgery [5]. Many retrospective studies have demonstrated the safety and efficacy of SBRT for oligometastases of various cancers, with local control up to 80% [6,7,8]. Preliminary randomized studies are promising. A significant improvement in progression-free survival was reported among patients with lung oligometastases who received SBRT following front-line systemic therapy compared to maintenance therapy or observation [9]. There was no increased grade 3–4 toxicity among patients who received SBRT. Thus, SBRT may be ideally suited for older patients with oligometastases, instead of surgery. As systemic therapy remains the standard treatment for stage IV disease, older cancer patients may not be able to tolerate chemotherapy due to frailty and/or pre-existing co-morbidities [1]. In addition, among older cancer patients with a good performance status, there may be reluctance among physicians to initiate chemotherapy, likely due to the fear of treatment toxicity [10,11]. Immunotherapy with checkpoint inhibitors (CPI) has been proposed as an effective systemic therapy for such patients [12]. A meta-analysis of two randomized studies for patients with metastatic non-small lung cancer receiving pembrolizumab suggests that adding radiotherapy may improve survival [13]. Thus, immunotherapy and SBRT may potentially improve survival for older cancer patients with oligometastases. However, the benefit of immunotherapy depends on the presence or absence of program death ligand 1 (PD-L1) receptors. Cancer patients with a high expression of PD-L1, defined as 50% or above, are likely to benefit the most from checkpoint inhibitors (CPI) independent of the tumor histology [14,15,16]. Even though PD-L1 is not a perfect biomarker, as tumors with little or no PD-L1 expression may still respond to CPI, the PD-L1 receptors may be increased by radiotherapy. Indeed, in vitro and in vivo studies of various tumors from different anatomic sites have reported a significant increase in PD-L1 expression following radiotherapy, which is in turn proportional to the radiotherapy dose [17,18,19,20,21,22]. Thus, could high dose of radiotherapy delivered precisely with SBRT induce an increased response to CPI for older cancer patients with oligometastases? We believe it could and propose a protocol using SBRT to initiate the tumor immune response which may be enhanced four to six weeks later with CPI. The protocol should be stratified to take into account potential differences in the response to immunotherapy with respect to gender or ethnicity. Older cancer patients, minorities, and women are currently under-represented in clinical trials. As an international organization dedicated to older cancer patients, the International Geriatric Radiotherapy Group (http://www.igrg.org, accessed on 26 November 2022) is investigating this hypothesis which may potentially improve the quality of care for such patients. As this is a new concept, we will briefly review the scientific rationale that supports our hypothesis.

## 2. Prevalence of PD-L1 Receptors among Different Types of Cancer

Program death ligands express on various different tumor types. In a study of 654 samples from 19 tumors of different anatomic sites, 89 (14%) were positive for PD-L1 [23]. The prevalence of PD-L1 receptors is increased with advanced disease stages and poorly differentiated tumors [24,25,26,27]. A meta-analysis of 9212 patients with epithelial cancer reported that expression of PD-L1 ranged from 44.5% to 51.6% and was dependent on ethnicity with a higher expression among Asians (52.3%) compared to Caucasians (32.7%) [28]. Even in a specific cancer, its expression varies among different histologies. PD-L1 expression may also vary depending on the anatomic site. For example, the prevalence of PD-L1 in melanoma ranges from 24% to 49% with the lowest rate among uveal melanoma (10%) [29,30]. In another example, PD-L1 expression in sarcoma ranges from 8.5% to 64.7% [31]. The highest expression of PD-L1 has been reported among Hodgkin and non-Hodgkin lymphoma, ranging from 30–70%, and may have been related to previous viral infections [32,33]. A similar high rate of PD-L1 expression, up to 70%, has been reported in glioma and in particular glioblastoma [34,35]. 

Thus, expression of PD-L1 is present in all tumor cells and presents an excellent opportunity to target them with CPI, especially when the tumor cells are strongly positive [14]. As an illustration, high PD-L1 expression has been correlated with an excellent survival from different tumor types after treatment with immunotherapy [36,37]. However, for those who have minimal or no expression of the biomarker, a high dose of radiotherapy may enhance the immunotherapy response through increased PD-L1 expression.

## 3. Effect of High Radiation Dose on the Tumor PD-L1 Expression

Radiotherapy induces inflammation within the tumor environment through a complex mechanism involving DNA damage, apoptosis, oxygen radical production, vascular damage, and autophagy [38]. As a result, a cascade of pro-inflammatory cytokines such as tumor necrosis factor α (TNF-α), Interleukin 6 (IL-6), Interferon γ (IFN-γ), and vascular endothelial growth factor (VEGF) is activated [39]. Death tumor cells express calreticulin on their cell surface which in turn activates dendritic cells to initiate phagocytosis of those cells. The activated dendritic cells reach the draining lymph nodes and activate lymphocytes, in particular CD8 T cells. This inflammatory change of the tumor micro-environment also results into the infiltration of activated CD8 T cells through liberation of chemokines such as CXCL16, and vascular cell adhesion molecule (VCAM)-1. Activated CD8 T cells acquire the ability to kill the tumor cells (cytotoxic T-lymphocytes) (CTLs) through apoptosis [40,41,42]. Thus, the effect of radiotherapy on the immune system plays an important role in tumor loco-regional control.

However, to avoid excessive damage to the normal organs, CD8 T cells also have PD-1 receptors which consist of 288 amino acid proteins and whose function is to attenuate the immune response [43]. Absence of PD-1 receptors often leads to diseases characterized by autoimmunity [44]. The binding of PD-1 receptors to PD-L1 receptors which are present on normal epithelial, hematopoietic, and endothelial cells, prevents destruction of those cells, and allows selective killing of pathogens such as virus or bacteria [29]. The presence of PD-L1 receptors on normal cells may be constitutional or inducible. As tumor cells also express PD-L1 receptors, they also avoid recognition and killing by CTLs. It has been reported that tumors with high PD-L1 expression are biologically aggressive and carry a poor prognosis [45,46]. Thus, PD-L1 is an effective mechanism for tumor cells to evade the patient’s immune system and to resist radiation-induced immune system activation. In vitro and in vivo studies support this hypothesis.

In NSCLC cell lines, radiotherapy induces an increase in PD-L1 expression through the phosphoinositide 3-kinase/AKT pathway [47]. In gastro-esophageal cancer cells with little or no PD-L1 expression at baseline, a high dose of radiotherapy (up to 16 Gy in a single fraction) produces significant increase in PD-L1 gene expression one week after treatment [48]. In a similar experiment using orthotopic mice which have developed oral cancer following injection of squamous cell cancer with minimal PD-L1 expression, radiotherapy induces significant PD-L1 expression proportional to the dose of radiation. The highest PD-L1 expression is observed following 24 Gy in a single fraction to the tumor. The rise of PD-L1 expression after radiotherapy is mediated through T-cell secretion of IFNℽ [49]. The role of IFNℽ in inducing PD-L1 expression has been demonstrated in sarcoma cell lines which are almost devoid of PD-L1. Introduction of IFNℽ in the culture milieu produces a significant increase in PD-L1 expression [50]. In other tumors with a high expression of PD-L1 at baseline such as bladder cancer, radiotherapy also enhances significantly PD-L1 expression proportional to radiation dose [17]. Other studies also corroborate the positive effect of radiotherapy on PD-L1 expression in different tumor cell types [17,51,52,53,54,55]. 

Clinical studies also support the role of high dose radiotherapy in inducing tumor production of PD-L1 and in particular, among tumors which do not express PD-L1 at diagnosis. In a study of 46 patients with locally advanced soft tissue sarcoma of the extremities who required preoperative radiotherapy, biopsy of the tumor performed before radiotherapy showed no PD-L1 expression. Following 50 to 50.4 Gy, 10.9% now demonstrated PDL-1 expression in the surgical specimen [56]. Radiotherapy alone or in combination with chemotherapy may also increase significantly PD-L1 expression in other tumors such as cervical or rectal cancer. Among 74 patients with locally advanced rectal cancer who required preoperative irradiation, PD-L1 expression was 15% and 50% before and after surgery, respectively [20]. The PD-L1 expression of cervical biopsy specimen taken before and after 10 Gy of chemoradiation was 5% and 52%, respectively [21]. Other studies also corroborate the important role of radiotherapy for inducing a strong (50% or above) PD-L1 expression in tumors which express PD-L1 at diagnosis [22,57,58]. Thus, a high dose of radiation if delivered safely may, in theory, induce or enhance tumor response to CPI irrespective of the tumor histology through PD-L1 expression.

## 4. The Role of SBRT in Patients with Oligometastases

Stereotactic body radiotherapy was initially introduced as an excellent modality to treat early stage non-small cell lung cancer (NSCLC) in older patients whose co-morbidities precluded surgery [5]. A high dose of radiotherapy is delivered with accurate precision while taking into account tumor motion to limit excessive radiation dose to the adjacent organs at risk (OAR) of radiation damage. Daily imaging with cone beam computer tomography (CBCT) scans or other imaging systems ensures perfect targeting before treatment. Thus, excellent local control and minimal complications have been reported in this vulnerable older patient population [59]. As a result of its safety profile, SBRT is becoming a popular treatment modality for patient with oligometastases instead of surgery [60].

In a study of 110 patients with pulmonary metastases who underwent pulmonary metastatectomy as their first choice or SBRT as an alternative if they were deemed unfit for surgery, local control and survival were similar in both groups. Five-year survival and two-year local control were 41% and 49%, and 90% and 94% for surgery and SBRT, respectively [61]. Even though the study was non-randomized, it did suggest that SBRT carries a similar outcome compared to surgery for oligometastases. In addition, other benefits of SBRT include treatment as an outpatient, low morbidity, and patient convenience since most of the treatment can be performed within a week in three to five treatments. Phase II randomized trials also suggest that compared to palliative treatment, SBRT confers a survival benefit in patients with oligometastases. Palma et al. [62] randomized 99 patients with oligometastases to SBRT (*n* = 66) and supportive care (*n* = 33). Median survival was 41 and 28 months for SBRT and the control group, respectively. Even though there was no subgroup analysis for older cancer patients, the median age was 69 and 67 years for the control group and SBRT group, respectively. Compared to other studies with the same concept, there was a higher mortality rate. For example, Gomez et al. [63] reported the results of 49 patients with NSCLC metastases aged from 43 to 83 years treated with SBRT (*n* = 25) or supportive care (*n* = 24). There was no reported death in the SBRT group. Even though older patients were included, there was no separate analysis on the influence of age on survival. Another phase II study corroborates this observation. A total of 54 patients with hormonal sensitive oligometastastic prostate cancer were randomized to observation (*n* = 18) or SBRT (*n* = 36). Disease progression at six months was 61% and 19% for the observation and SBRT groups, respectively (*p* < 0.005). There was also significantly less new metastatic disease in the SBRT arm, suggesting that radiotherapy may have prevented new disease through a systemic effect [64]. There was also no grade 3 adverse event reported for patients who had radiation. The median age of the patients was 68 and ranged from 61 to 70 years. Thus, the inclusion of older cancer patients in those studies highlights SBRT’s safety.

Two other phase II randomized studies also highlight the safety and efficacy of SBRT when combined with systemic therapy. Among 29 NSCLC patients with oligometastases without EGFR-targetable or ALK-targetable mutation who finished induction chemotherapy, 14 had SBRT and maintenance chemotherapy, and 15 had maintenance chemotherapy only. Progression-free survival was 9.7 months and 3.5 months for the combined and control arm, respectively (*p* = 0.01). There was no difference in toxicity between the two arms [65]. In 133 NSCLC patients with EGFR-mutated adenocarcinoma and oligometastases, the addition of SBRT (*n* = 68) significantly improved survival when they also received tyrosine kinase inhibitor (TKI) compared to TKI alone. Median survival was 25.5 and 17.4 months for the combined and the control group, respectively (*p* < 0.001). However, grade 3–4 pneumonitis was higher (6%) for the experimental arm [66]. As the median age for patients treated with SBRT and TKI was 67 years, this study corroborates the safety of SBRT when combined with a radiosensitizer. A meta-analysis of SBRT for oligometastases of various histology and anatomic sites also corroborates the additional survival benefit and safety of SBRT when combined with systemic therapy [67]. The efficacy of SBRT is linked to the highest dose of radiotherapy that can be delivered effectively. In a phase III randomized study of 117 patients with oligometastases, 59 received a high single dose (24 Gy) and 58 had fractionated SBRT (9 Gy times 3 fractions). Three-year local recurrences and cumulative distant metastases were 5.8% and 22% and 5.3% and 22.5% for the high single dose arm and the fractionated arm, respectively [68]. The increased biological equivalent dose (BED) achieved in the high single dose arm (81.6 Gy) may have been responsible for the increased local control while mitigating the risk of distant metastases. This short fractionation may be very convenient for older cancer patients due to their limited mobility and difficulty with transportation [69,70]. In cancers that are resistant to radiotherapy such as renal cell cancer and melanoma, high rates of local control up to 90% have been reported for oligometastases due to the high BED [71,72]. The combination of SBRT with TKI for renal cell oligometastases is also well tolerated and delays disease progression by one year in older cancer patients [73]. Another study corroborates the safety and efficacy of SBRT when combined with systemic therapy in older cancer patients. Among 254 patients aged 65 years old or above, who had their primary cancer controlled but developed oligometastases later, 156 received systemic therapy alone and 86 combined with radiotherapy. No patient in the combined therapy group developed grade 3–4 complications. Median survival was significantly improved with the addition of SBRT and was 25 months and 16 months with and without radiotherapy, respectively [74]. Thus, SBRT is safe and effective among older cancer patients with oligometastases receiving systemic therapy. Table 1 summarizes the efficacy of SBRT in patients with oligometastases from various tumor types.

## 5. The Role of Immunotherapy for the Treatment of Oligometastases

Immunotherapy has been used successfully for the treatment of oligometastases. In a study of 59 patients with NSCLC and oligometastases, neoadjuvant CPI and chemotherapy have been used to induce tumor shrinkage for primary tumor resection and metastasectomy followed by consolidation CPI. Overall two-year survival was 87.2% [75]. Another study also corroborates the success of neoadjuvant immunotherapy either alone or combined with chemotherapy for oligometastatic NSCLC with a complete pathologic rate of 54% [76]. Among 35 patients with oligometastatic melanoma, neoadjuvant ipilimomab and nivolumab followed by surgery and adjuvant nivolumab, a high complete pathological response rate of 55% has been reported [77]. In 259 patients with metastatic gastric or gastroesophageal junction cancer who failed previous therapy, pembrolizumab was associated with an overall response rate of 11.6%. The response rate for PD-L1 positive and negative tumors were 22.7% and 6.4%, respectively [78]. Thus, the benefit of immunotherapy alone is best when the tumor expresses PD-L1 or in tumors with high PD-L1 expression such as melanoma. If SBRT is added to immunotherapy, local control and survival may improve due to the increased PD-L1 expression following SBRT.

## 6. Discussion about the Potential Role of Immunotherapy in the Treatment of Oligometastases in Older Cancer Patients

In theory, immunotherapy and SBRT should work in synergy to improve tumor cell killing. The combination of CPI and high dose of radiation has been reported to increase tumor control compared to either modality alone in animal experiments [79,80,81]. For example, in murine hepatocellular carcinoma, high dose radiotherapy increases PD-L1 expression in the tumor which is then exposed to CPI, leading to improved survival of the rats which received radiotherapy and CPI, compared to those treated with radiotherapy alone or CPI alone [82]. A similar experiment involving NSCLC implanted in rats corroborated the synergy between radiotherapy and CPI when radiotherapy was initiated first to enhance the effect of immunotherapy [83]. Interestingly, in another experiment, the benefits of high dose local radiation (30 Gy) also enhanced the survival of the rats which improved further with the addition of CPI suggesting an abscopal effect of radiation through activation and mobilization of CD8 T cells [84,85]. Abscopal effect is defined as the systemic effect outside of the irradiated volume. This interaction between radiotherapy and immunotherapy is complex and postulated through increased antigen presentation, increasing T cells recruitment, up-regulating PD-L1 receptor, and alteration of the tumor microenvironment [85].

Clinical studies also support the use of immunotherapy with SBRT for patients with oligometastases. Preliminary data suggests that the combination of immunotherapy and radiotherapy is safe. Among the nine patients with NSCLC oligometastases treated with durvalumab and tremelimumab and an SBRT dose ranging from 30 to 50 Gy in five fractions, no death was observed [86]. Another study on NSCLC oligometastases corroborates the safety of the combined treatment. In 100 patients with metastatic NSCLC treated with SBRT and various dose of pembrolizumab or pembrolizumab alone, no death was reported. Interestingly, even though there was no difference in the response rate between the two groups, patients with no PD-L1 expression (*n* = 19) seemed to benefit the most from radiotherapy. The median progression-free survival was 20.8 and 4.6 months with and without radiotherapy, respectively [87]. Even though the patient number is small, the benefit observed by the addition of SBRT suggests that radiotherapy may have induced PD-L1 expression for those tumors which rendered them more sensitive to pembrolizumab. In a review of 371 patients who had immunotherapy and SBRT for oligometastatic NSCLC oligometastases, survival was improved for those who received immunotherapy more than three weeks after radiotherapy [88]. We postulate that the delayed time after irradiation may allow the tumor to increase its PD-L1 expression and may have accounted for the improved survival compared to those who were treated for a shorter period of time. Indeed, in a prospective trial of 45 patients with NSCLC oligometastases who received pembrolizumab four to eight weeks after SBRT, median progression-free survival (PFS) was signicantly improved. Median PFS was 19.1 months and 6.6 months for the study group and historical control, respectively [89]. The synergy between SBRT and immunotherapy for oligometastatic NSCLC was highlighted in another study. Of 152 patients enrolled in the study, median PFS was 13.8 and 8.9 months for the combined CPI-SBRT and CPI alone, respectively (*p* = 0.03). In a subgroup analysis of patients with known PD-L1 status, patients with no PD-L1 expression seemed to benefit the most from the addition of radiotherapy [90]. The most compelling evidence for the benefit of radiotherapy for patients with oligometatastic NSCLC and negative PD-L1 expression has been reported in a phase II randomized trial comparing pembrolizumab either alone (*n* = 40) or after SBRT (*n* = 36). There was no significant difference in survival, PFS, response rate, and toxicity between those two groups. However, among patients with no PDL-1 expression, response rate and PFS was significantly improved [91]. Even though this is a subgroup analysis with a small number of patients, the data is intriguing and merits further investigation.

The combination of immunotherapy and SBRT is also safe and effective for other types of cancer. Among 30 patients with oligometastatic renal cancer which is traditionally resistant to chemotherapy and radiotherapy, two-year survival and PFS was 74% and 45%, following SBRT and pembrolizumab, respectively [92]. A total of 83% of the patients achieved local control. Grade 3 side effects occurred in four patients (13%). In another radio-resistant tumor such as melanoma, the addition of SBRT to CPI has produced prolonged remission among patients who had multiple recurrences [93]. In patients with oligometastatic bladder cancer, preliminary results of the combined modality have been reported to be promising [94]. Thus, preliminary evidence suggests that the combination of SBRT and immunotherapy is feasible for patients with oligometastases, but needs further investigation.

Older cancer patients with oligometastases may be the best candidates for the combined treatment. Metastasectomy may not be an option for those patients with multiple co-morbidities due to a high mortality rate [3,4,95]. Chemotherapy may not be tolerated as well for the same reason [9]. Tumor ablation with SBRT is effective and well tolerated for all age groups. In addition, there may be a synergy with immunotherapy through a complex process which may be beneficial for all patients, regardless of their initial PD-L1 status. Immunotherapy following SBRT may be the best option for older cancer patients to achieve long-term remission. Younger but physiologically frail patients may also benefit from the protocol. Thus, the IGRG proposes a new algorithm to be tested in future prospective clinical trials.

## 7. Incorporation of Frailty as a Risk Factor for Toxicity into Future Clinical Trials and the Important Role of the Interdisciplinary Team in Patient Management during the Trial

Frailty, defined as an accumulative decline in physical reserve, is a serious issue that affects patient tolerance to treatment regardless of age. Physiologically frail young patients experienced a higher rate of hospitalization and treatment incompletion compared to those who are fit [96]. Thus, comprehensive assessment of frailty is often conducted through screening questionnaires such as the comprehensive geriatric assessment (CGA). However, the CGA is a cumbersome test to conduct for all patients if the trial includes a large number of patients. Other tests, such as the G8 screening test, are more simple and easier to conduct. A G8 score of 14 or lower indicates a poor health status and the need to undergo a comprehensive assessment through the CGA. In addition, there was a correlation between a low G8 score and toxicity among older patients with early stage NSCLC undergoing hypofractionated radiotherapy [97]. The correlation between the G8 score and tolerance to SBRT for older patients with oligometastases was also corroborated in another study [98]. There was minimal toxicity and good local control for older cancer patients with oligometastases who were deemed unfit for systemic therapy when a fractionation of 7 Gy times 5 (BED = 59.5 Gy) was used [98,99,100]. Thus, a BED around 59.5 Gy and screening with G8 seem to be reasonable criteria for physiologically frail patients with oligometastases. However, a pooled analysis of two randomized studies with a higher BED (>100 Gy) and immunotherapy for metastatic NSCLC did not report any increase in toxicity or decreased survival among patients who received SBRT and immunotherapy [13]. Thus, the optimal dose of radiotherapy and immunotherapy need to be determined in future clinical trials.

Older cancer patients with oligometastases should be evaluated by a multi-disciplinary team which includes oncology specialists, geriatricians, nutritionists, and social workers to determine the patients’ physical, mental, and social status [70]. This multidisciplinary approach which incorporated a geriatrician in the team managing older cancer patients has been touted as effective to improve patient care and quality of life [101]. Physiologically frail patients frequently require many medications which may lead to toxicity. Those who are malnourished may not tolerate treatment. Transportation issues and isolation may lead to depression and treatment interruption. Thus, we advocate a multidisciplinary approach to monitor patients during and after treatment which may be facilitated with telemedicine [102]. Biomarkers and oncogenomics could be incorporated to assess the clinical outcomes and to identify patients most likely to respond to treatment and treatment toxicity [103]. Preliminary evidence suggests that clinical response and immunologic toxicity may be dependent on ethnicity [104,105]. As tumor PD-L1 expression may differ among different ethnic groups, it may be the underlying cause for the various immune responses [28]. Unfortunately, minority, women, and older cancer patients are not frequently represented in current clinical immunotherapy trials to investigate this hypothesis [106]. As our IGRG network includes over 1100 institutions in 127 countries, recruitment of those patients may not be an issue [70]. 

## 8. Potential Protocol Proposed by the International Geriatric Radiotherapy Group

Chronologically older cancer patients, defined as 70 years of age or above, and physiologically frail younger patients with oligometastases, defined as five or fewer, will be recruited into the study. All patients will undergo a G8 screening survey. Those with a score of 15 or above will be defined as fit. Those with a score of 14 or less will undergo a comprehensive geriatric assessment with the CGA survey to assess the severity of the frailty. Fit younger patients will be excluded from the study. Patients with brain metastases, uncontrolled loco-regional disease, a history of prior malignant tumor, or autoimmune disease are also excluded. All metastatic diseases should receive a dose of at least 59.5 Gy RBE. Immunotherapy should be started four to six weeks after completion of SBRT.

The primary objective of the study is to test the hypothesis that SBRT and immunotherapy may improve survival and disease-free survival for older patients and physically frail patients with oligometastases as compared to the institution standard of care of no therapy, SBRT only, or immunotherapy only.

The secondary objective of the study is to determine whether toxicity may be reduced with a standard dose of immunotherapy every three weeks or every six weeks.

**Figure 1:**
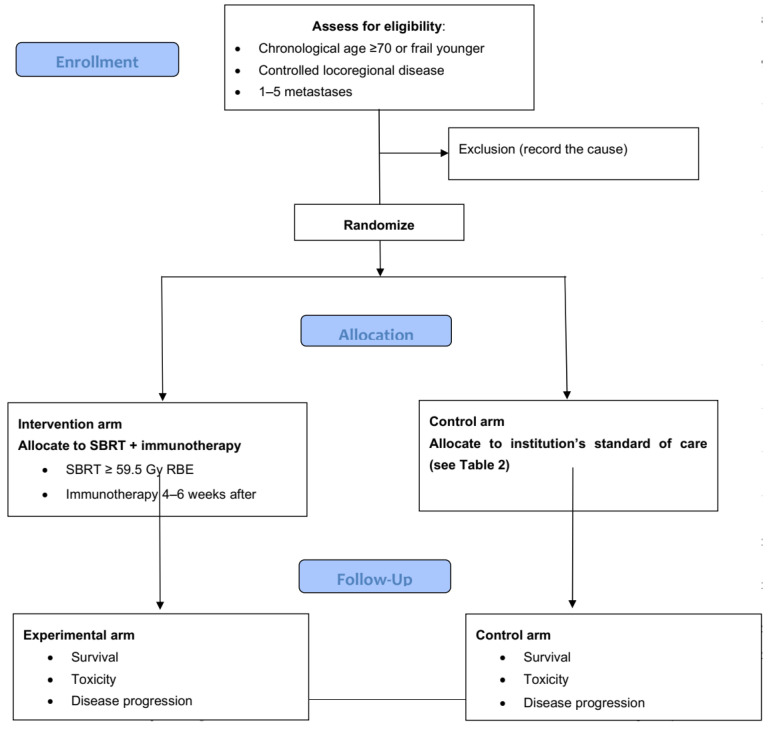
Flowchart illustrating the selection process of older cancer patients or younger frail patients with oligometastases.

Patients will be stratified based on gender, PD-L1 expression, radiotherapy dose level, immunotherapy dose, and type of primary tumor. Table 2 and Figure 1 summarize the design of the study.

Patients will be monitored with an interdisciplinary team throughout the study. Telemedicine will be employed to minimize transportation issues and exposure to the coronavirus.

## 9. Conclusions

Stereotactic body radiotherapy may induce a favorable immune response to CPI through the tumor production of PD-L1. Thus, a protocol using SBRT first followed four to six weeks later with immunotherapy may improve survival and disease-free survival of older cancer patients or frail younger patients with oligometastases while minimizing toxicity. This hypothesis should be tested in future prospective studies. We advocate a multidisciplinary team to conduct those studies due to the complexity of managing frail patients with multiple co-morbidities.

## Figures and Tables

**Table 1:** Studies summarizing the efficacy of stereotactic body radiotherapy for oligometastases of various tumor types. SBRT, stereotactic body radiotherapy; S, surgery; SC, supportive care; C, chemotherapy; PFS, progression-free survival; tki, tyrosine kinase inhibitor; ST, systemic therapy; NS, not specified.

Study	Patient No.	Primary	No. of Metastases	Metastatic Site	Local Control	Survival	Complications
Widder et al. [61]	42 SBRT	Multiple	5 or less	Lung	94% (2-year)	49% (5-year)	NS
	68 S				90% (2-year)	41% (5-year)	
Palma et al. [62]	66 SBRT	Multiple	5 or less	Multiple sites	75% (2-year)	Median: 41 m	4.5% death
	33 SC				49% (2-year)	Median: 28 m	No death
Gomez et al. [63]	66 SBRT	Lung	3 or less	Multiple sites	NS	Median: 41.2 m	No gr. 3–4
	33 SC					Median: 17 m	
Phillips et al. [64]	36 SBRT	Prostate	3 or less	Multiple sites	98.9%	NS	No gr. 3–4
	18 SC						
Iengar et al. [65]	14 SBRT+C	Lung	5 or less	Multiple sites	100% (1-year)	Median PFS: 9.7 m	28.5% gr. 3–4
	15 C				46.6% (1-year)	Median PFS: 3.5 m	20% gr. 3–4
Wang et al. [66]	68 SBRT+tki	Lung	5 or less	Multiple sites	91.2% (1-year)	Median S: 25.5 m	35.4% gr. 3–4
	65 tki				55.4% (1-year)	Median S: 17.6 m	39.9% gr. 3–4
Franceschini et al. [72]	31	Melanoma (skin)	3 or less	Multiple sites	96.8% (1-year)	41% (1-year)	No gr. 3–4
Cheung et al. [73]	37 SBRT+tki	Kidney	5 or less	Multiple sites	93% (1-year)	92% (1-year)	No gr. 3–4
Hu et al. [74]	86 SBRT+ST	Multiple	5 or less	Multiple sites	NS	Median S: 25 m	3.5% gr. 3
	156 (ST)					Median S: 16 m	

**Table 2:** Proposed randomized study to test the hypothesis that stereotactic body radiotherapy and immunotherapy may improve survival of older patients with oligometastases.

Treatment	Immunotherapy	No Immunotherapy
SBRT	Experimental arm = SBRT + immunotherapy	Standard chosen by institution = SBRT only
No SBRT	Standard chosen by institution = immunotherapy only	Standard chosen by institution = No SBRT + No immunotherapy

## Data Availability

Not applicable.
